# Molecular Basis of Aquaporin-7 Permeability Regulation by pH

**DOI:** 10.3390/cells7110207

**Published:** 2018-11-10

**Authors:** Andreia F. Mósca, Andreia de Almeida, Darren Wragg, Ana P. Martins, Farzana Sabir, Stefano Leoni, Teresa F. Moura, Catarina Prista, Angela Casini, Graça Soveral

**Affiliations:** 1 Research Institute for Medicines (iMed.ULisboa), Faculty of Pharmacy, Universidade de Lisboa, 1649-003 Lisboa, Portugal; andreiafbm@ff.ulisboa.pt (A.F.M.); martinsap@ff.ulisboa.pt (A.P.M.); fsabir@isa.ulisboa.pt (F.S.); teresa@ff.ulisboa.pt (T.F.M.); 2 Dept. Bioquímica e Biologia Humana, Faculty of Pharmacy, Universidade de Lisboa, 1649-003 Lisboa, Portugal; 3 School of Chemistry, Cardiff University, Park Place, Cardiff CF10 3AT, UK; DeAlmeidaA@cardiff.ac.uk (A.d.A.); WraggDD@cardiff.ac.uk (D.W.); LeoniS@cardiff.ac.uk (S.L.); 4 Tumour MicroEnvironment Group, Division of Cancer and Genetics, School of Medicine, Cardiff University, Tenovus Building, Cardiff CF14 4XN, UK; 5 LEAF, Linking Landscape, Environment, Agriculture and Food, and DRAT, Dept. de Recursos Biológicos, Ambiente e Território, Instituto Superior de Agronomia, Universidade de Lisboa, Tapada da Ajuda, 1349017 Lisboa, Portugal; cprista@isa.ulisboa.pt; 6 Faculdade de Ciências e Tecnologia, Universidade Nova de Lisboa, 2829-516 Caparica, Portugal

**Keywords:** aquaporin, aquaglyceroporin, AQP7, pH, yeast, regulation, water and glycerol permeability

## Abstract

The aquaglyceroporin AQP7, a family member of aquaporin membrane channels, facilitates the permeation of water and glycerol through cell membranes and is crucial for body lipid and energy homeostasis. Regulation of glycerol permeability via AQP7 is considered a promising therapeutic strategy towards fat-related metabolic complications. Here, we used a yeast *aqy*-null strain for heterologous expression and functional analysis of human AQP7 and investigated its regulation by pH. Using a combination of in vitro and in silico approaches, we found that AQP7 changes from fully permeable to virtually closed at acidic pH, and that Tyr135 and His165 facing the extracellular environment are crucial residues for channel permeability. Moreover, instead of reducing the pore size, the protonation of key residues changes AQP7’s protein surface electrostatic charges, which, in turn, may decrease glycerol’s binding affinity to the pore, resulting in decreased permeability. In addition, since some pH-sensitive residues are located at the monomer-monomer interface, decreased permeability may result from cooperativity between AQP7’s monomers. Considering the importance of glycerol permeation via AQP7 in multiple pathophysiological conditions, this mechanism of hAQP7 pH-regulation may help the design of selective modulators targeting aquaglyceroporin-related disorders.

## 1. Introduction

Aquaporins (AQPs) are a family of small membrane proteins expressed in almost every organism and tissue type and involved in the bidirectional transfer of water and small solutes across cell membranes in response to osmotic and hydrostatic pressure or concentration gradients. Mammalian AQPs (AQP0-12) are grouped according to their primary structure and permeability. The aquaglyceroporin subfamily (AQP3, 7, 9, 10) facilitates permeation of glycerol and other small solutes in addition to water [1]. Due to their selective permeability, aquaglyceroporins have important roles in glycerol accumulation and metabolism in tissues, such as skin, fat, and liver [2,3,4], and are considered to be implicated in obesity and metabolic-related complications, such as metabolic syndrome [5,6].

Structural studies of AQPs from different species revealed a common homotetrameric assembly in the plasma membrane [7,8], assumed to be preserved among all AQP family members, with each monomer functioning independently as a water or glycerol channel. Interestingly, AQPs show a highly conserved hourglass-shaped internal pore, which results in a steric size-excluding barrier for solutes. This barrier consists of three (in aquaglyceroporins) or four (in strict aquaporins) amino acid residues, of which one is a highly conserved arginine and at least one has an aromatic side chain. Therefore, this constriction site is named the aromatic/arginine selectivity filter (ar/R SF) [9]. A second barrier is formed in the centre of the pore, where the ends of two internal semi-helices meet and consist of motifs of NPA (asparagine-proline-alanine) in each helix, considered to be the signature of the AQP family. However, the NPA motif is not always conserved across isoforms of the same or different species, where the proline and alanine may be substituted, but the two asparagine amino acid residues are highly conserved. The sidechains of the two asparagines create a positive dipole moment in the pore, preventing protons and other charged species from crossing the pore [10,11,12].

Like many other channels and transporters, AQPs can be subjected to regulation, either by transcriptional/translational mechanisms [13], protein trafficking [14], or by channel short-term regulation, also known as gating. This is often achieved by mechanisms directly affecting the channel conformation after its insertion in the plasma membrane, which impacts its permeability [15]. Indeed, it has been shown that various eukaryotic water-selective aquaporins are gated by phosphorylation [16,17], pH [17,18,19], divalent cations [20,21], or membrane surface tension [22,23,24,25]. Less is known about the regulation of aquaglyceroporins and glycerol permeability. With AQPs being emerging protein targets for drug development [26], a better understanding of human aquaglyceroporin regulation would facilitate the identification of new putative modulators with potential therapeutic applications for human health [27,28].

Notwithstanding the lack of knowledge in aquaglyceroporin regulation, a few studies have shown gating of AQP3 by pH, copper, and nickel [29,30,31,32]. Recently, we have fully characterized AQP3-pH gating using human red blood cells and yeast cells expressing the rat AQP3 isoform [32]. Through computational studies, we investigated the molecular mechanisms responsible for the pH-dependent closure/opening of hAQP3 and disclosed important structural features that can be targeted for modulation.

In this study, we investigated pH regulation of human AQP7, the main aquaglyceroporin expressed in human adipocytes and particularly relevant to assure efficient glycerol fluxes in healthy adipose tissue, being involved in obesity and fat-related metabolic complications [4,5,6,33]. By expressing hAQP7 in our optimized yeast expression system [15], we were able to detect and characterize water and glycerol permeability corroborating the hAQP7 channel function. Moreover, channel permeability was confirmed by inhibition with Auphen–[Au(phen)Cl_2_]Cl (phen = 1,10-phenanthroline)—a gold(III) compound reported by us as a potent inhibitor of human aquaglyceroporins, AQP3 and AQP7, in their native expression systems [34,35]. Using a combination of in vitro and in silico techniques, we investigated the mechanisms involved in hAQP7-pH regulation at a molecular level using site-directed mutagenesis combined with molecular modelling and molecular dynamics (MD) approaches. Knowledge of hAQP7 regulation can contribute to understanding the roles of this isoform in physiology and pathology, helping the development of aquaglyceroporin-targeted therapies.

## 2. Materials and Methods

### 2.1. Strains, Plasmids, and Growth Conditions

Human AQP7 (hAQP7) cDNA was PCR-amplified from the pWPi-DEST-AQP7 plasmid [36] and C-terminally fused to a green-fluorescent protein (GFP) of the centromeric plasmid, pUG35 [37]. *Escherichia coli* DH5α [38] was used as a host for routine propagation and purification of the plasmids with a GenElute^TM^ Plasmid Miniprep Kit (Sigma-Aldrich, St. Louis, MO, USA). *E. coli* transformants were maintained and grown in Luria-Bertani broth (LB) at 37 °C, with ampicillin (100 µg/mL) [39].

For functional studies, *Saccharomyces cerevisiae*, 10560-6B MATα leu2::hisG trp1::hisG his3::hisG ura352 aqy1D::KanMX aqy2D::KanMX (YSH1770, further indicated as *aqy*-null) was used as a host strain for heterologous expression of hAQP7. Yeast cultures were grown at 28 °C with orbital shaking in YNB (yeast nitrogen base) without amino acids (DIFCO), 2% (*w*/*v*) glucose supplemented with the adequate requirements for prototrophic growth [40].

### 2.2. Heterologous Expression of hAQP7 in S. cerevisiae

*E. coli* DH5α was transformed with pWPi-DEST_lnAQP7 and used for propagation of the plasmid. Plasmidic DNA was isolated and purified. Specific primers modified to incorporate restriction sites for *Spe*I (bold) and *Cla*I (bold) were designed and used for amplification of hAQP7 cDNA (Table S1). PCR amplification was carried out in an Eppendorf thermocycler with proofreading *Taq* Change DNA polymerase (NZYTech). A temperature gradient PCR was performed to determine the optimum annealing temperature. The amplified product was digested with *Spe*I and *Cla*I (Roche Diagnostics^®^) and purified using Wizard^®^ SV Gel and PCR Clean-Up System kit (Promega). The purified product was cloned into the corresponding restriction sites of pUG35 digested with the same restriction enzymes, behind the MET25 promoter and in frame with GFP sequence and CYC1-T terminator, using T4 DNA Ligase (Roche, Basel, Switzerland). Cloning was performed according to standard protocols [39] to construct the expression plasmid, pUG35-hAQP7. The plasmid was used to transform DH5α *E. coli* strain, propagated and subjected to extraction and purification. Fidelity of constructs and correct orientation was verified by PCR amplification, restriction analysis, and DNA sequencing. Agarose gel electrophoresis and restriction site mapping were performed according to standard methods [39,41].

Transformation of the *aqy*-null strain with pUG35-hAQP7 was performed by the lithium acetate method described in [41]. The same strain was also transformed with the empty pUG35 vector (which does not contain hAQP7 cDNA) to be used as a control (further indicated as control cells). Transformants were selected on YNB medium without uracil as an auxotrophic marker.

### 2.3. Subcellular Localization and Membrane Abundance Analysis by Fluorescence Microscopy

For subcellular localization of GFP-tagged hAQP7 in *S. cerevisiae*, yeast transformants in the mid-exponential phase were observed with a Zeiss Axiovert 200 fluorescence microscope, at 495 nm excitation and 535 nm emission wavelengths. Fluorescence microscopy images were captured with a digital camera (CoolSNAP EZ, Photometrics, Huntington Beach, CA, USA) and using the Metafluor software (Molecular Devices, Sunyvale, CA, USA) (Figure S1). hAQP7 membrane expression was measured at pH 7.4 and pH 5.1 by measuring GFP-protein fluorescence intensity according to [14,42] and as previously reported by us [17]. A linear intensity profile across the cell membrane was generated and analyzed using the software, ImageJ (https://imagej.net). The intensity profile along the line path from at least 30 cells in each experimental condition (*n* = 3) was recorded, and for each cell, three profile lines were taken. The background intensity along the same distance was measured and subtracted from the peak fluorescence intensity over each line, and the obtained difference divided by the maximal fluorescence to calculate the relative membrane expression (RME).

### 2.4. Site-Directed Mutagenesis

PCR-based site-directed mutagenesis was performed to introduce point mutations into wild-type hAQP7 cDNA, where Tyr135, His140, and His165 were replaced with Alanine residues (Ala), using the recombinant plasmid, pUG35-hAQP7, as a template. The mutagenic primers used in this study and the respective substitutions introduced (underlined) are listed in Table S1. Each mutation at the corresponding position was confirmed by DNA sequencing, and further PCR reactions were performed to create double mutations (Y135A+H165A and H140A+H165A). All PCR mutagenized products were purified, digested, and cloned in pUG35, and yeast cells were transformed as described above.

### 2.5. Permeability Assays

Yeast transformants were cultured up to OD_640nm_ ≈ 1 (corresponding to 1 × 10^7^ cells/mL) and harvested by centrifugation (5000× *g*; 10 min; 4 °C). Afterwards, cells were washed and re-suspended in ice-cold sorbitol (1.4 mol/L) K^+^-citrate buffer (50 mmol/L, pH 7.4), up to a concentration of 0.33 g·mL^−1^ wet weight and kept on ice for at least 90 min. Prior to the osmotic challenges, the cell suspension was pre-loaded with the non-fluorescent precursor, 5-and-6-carboxyfluorescein diacetate (CFDA, 1 mM for 10 min at 30 °C), that is cleaved intracellularly by nonspecific esterases and generates the impermeable fluorescent form, carboxyfluorescein, known to remain in the cytoplasm [43]. Equilibrium cell volumes were obtained by loading cells with CFDA under a fluorescent microscope equipped with a digital camera as previously described [43]. Cells were assumed to have a spherical shape with a diameter calculated as the average of the maximum and minimum dimensions of each cell.

Stopped-flow was used to monitor cell volume changes of cells loaded with a concentration-dependent self-quenching fluorophore [43]. Experiments were performed on a HI-TECH Scientific PQ/SF-53 stopped-flow apparatus, which has a 2 ms dead time, temperature controlled, and interfaced with an IBM PC/AT compatible 80386 microcomputer. Experiments were performed at 23 °C. Five runs were usually stored and analysed in each experimental condition. In each run, 0.1 mL of cell suspension (1:10 dilution in resuspension buffer) was mixed with an equal amount of iso- (baseline) or hyperosmotic solutions (of sorbitol or glycerol) of 1.25 tonicity ((*Λ= (osm_out_)_∞_/(osm_out_)_o_*), 350 mmol/L gradient). The fluorophore was excited using the light source with a 470 nm interference filter, detected using a 530 nm cut-off filter, and the changes in fluorescence due to the carboxyfluorescein fluorescence quenching were recorded.

For inhibition experiments, cell suspensions equilibrated in an isotonic solution (sorbitol 1.4 mol/L) were incubated with CFDA in the absence or presence of Auphen (70 μmol/L), at room temperature for 30 min at the selected pH.

To characterize the pH-dependence of hAQP7 permeability, yeast transformants were incubated in an isotonic solution (sorbitol 1.4 mol/L) with different pH (varying from 5.0 to 7.5) for at least 90 min. In these conditions, cells deprived of carbon sources and incubated on ice for a long period are considered in starvation and unable to maintain an internal pH gradient [44]. After the incubation with the fluorescence probe, stopped-flow experiments were performed at 23 °C for both water and glycerol transport at different external pH values.

### 2.6. Calibration of the Fluorescence Signals into Relative Volume

The fluorescence traces obtained were corrected by subtracting the baseline trace that reflects the bleaching of the fluorophore. The calibration of the resulting traces for the two strains followed our previous strategy [22], where a linear relationship between relative volume and F was obtained (*v_rel_* = *a F/F0 + b*); the values of *a* and *b* were estimated individually for each sorbitol osmotic shocks, considering the initial and final fluorescence values and the correspondent relative volumes obtained previously by our group for the same tonicity shock [22,43]. These values were then used for the calibration of the traces in the glycerol osmotic shock performed under the same experimental conditions, tonicity, and temperature.

### 2.7. Permeability and Activation Energy Evaluation

The experimental protocols to assess aquaporin function were designed to keep the membrane surface tension to a minimum, in order to maintain aquaporin activity at its maximum, as previously found in our laboratory [22]. This was accomplished by equilibrating cells in 1.4 mol/L sorbitol solution (considering sorbitol as a non-diffusible solute) followed by the application of low tonicity hyperosmotic shocks (*Λ =* 1.25, 350 mmol/L gradient) with sorbitol or glycerol.

For this purpose, the calibrated experimental curves, *v_rel_*, were fitted to their theoretical curves, considering the water and glycerol fluxes and the resulting changes in cell volume and intracellular concentrations of solutes. Optimization of permeability values was accomplished by numerical integrations using a mathematical model implemented in the Berkeley Madonna software (http://www.berkeleymadonna.com/).

The activation energy (*E_a_*) of water and glycerol transport was evaluated by performing permeability assays at temperatures ranging from 7 to 38 °C. *E_a_* values were obtained from the slope of Arrhenius plots (lnP_f_ or lnP_Gly_ as a function of 1/T).

### 2.8. Molecular Modeling and Analysis

The 3D structure of hAQP7 was obtained by homology modelling using Molecular Operating Environment (MOE 2012.10) (CCG 2012) [45]. The choice of a template structure was based on the sequence identity between hAQP7 and the sequence of the AQPs with available resolved structures from human, bacteria, and *Plasmodium falciparum* (UniProt 2013 codes O14520, C8TK05, and Q8WPZ6, respectively). The isoform that has the highest sequence similarity with hAQP7 is the bacterial isoform, Glycerol Facilitator (GlpF) (24.5% identity), which was then chosen as a template structure. Three resolved structures for bGlpF, crystallized either with or without glycerol and solved by X-ray diffraction, were retrieved from the Protein Data Bank [46]. Among them, the template was selected that had the best resolution (2.2 Å, pdb 1FX8). The biological assembly, containing the tetramer structure, was prepared and protonated at pH 7.0 under forcefield Amber12EHT. Thus, the tetrameric form of the human AQP7 model was built: 50 intermediate models were generated and averaged to obtain the final homology model.

The obtained model was checked for more realistic rotamers of side chains in the regions of ar/R SF and NPA, by comparison with the available crystal structures of all the other AQP isoforms (pdb codes 1H6I, 36D8, 3D9S, 1RC2, 1LD1, and 3C02). The structure was protonated at pH 7.0 and an energy minimization refinement was performed, also under the Amber12EHT force field, during which the Cα atoms were fixed. After identification of the residues of interest for the mechanism of pH gating, the same energy minimization procedure was used to further refine them. The refined homology model of hAQP7 was protonated using the PROPKA 3.1 package [47] at pH 5.0 and 7.0. Electrostatic surfaces were generated using the Adaptive Poisson-Boltzmann Solver (APBS) [48] plugin in Chimera [49].

### 2.9. Molecular Dynamics

Two model systems, using the above-described homology model, were produced using the PDB2PQR Server (version 2.0.0) [47], protonated at pH 7.0 and pH 5.0. The molecular systems consisted of the protonated tetrameric models of the AQP7 within a double layer of 175 palmitoyl-oleoyl-phosphatidyl-coline (POPC) lipid using the charm-gui online server [50]. Four glycerol molecules were placed into the system, one above each pore entrance on the extracellular side, at an approximate distance of 30 to 35 Å. To evaluate glycerol uptake, the system was solvated with 33151 (pH 7.0) and 33098 (pH 5.0) TIP3P water molecules and used a modified amber99sb-ildn forcefield, with the parameters for glycerol generated by the Automated Topology Builder and Repository (ATb, version 2.2) website using the B3LYP/6-31G* basis set [51].

All simulations were run using the GROMACS 5.1.2 simulation software [52] with a 2 fs time step. The particle-mesh Ewald method was used for calculating electrostatic interactions. The verlet cut-off scheme with a cut-off distance of 1.4 nm was used for short-range repulsive and attractive interactions and Lincs was used to constrain all bond lengths. Nose-Hoover temperature coupling was used to maintain the temperature of the system (т = 0.5 ps) at 310 K. The Parrinello-Rahman algorithm was used to maintain the pressure of the system at 1 bar with a coupling constant of т = 1.0 ps. Simulations were equilibrated for 100 ps before production.

The four individual glycerol molecules were defined in the index and coupled in the pull code (e.g., gly_1 to chain_A). A total of 10 MD simulations (5 for the pH 7.0 system and 5 for the pH 5.0 system) were run using the direction COM pull procedure, in each case applying a separate yet equal harmonic restraint force to each solute molecule of 100 kJ mol^−1^nm^2^ with a rate of 0.02 nm ns^−1^ along the z-axis in the direction of the intracellular space. Simulations were run for 250,000 steps or 500 ps, with a timestep of 2 fs. Each pore radius was calculated using the Hole 2.0 program [53], which determines the internal surface based on atomic van der Waals radii. Snapshots were taken at 5 equal intervals throughout the trajectories, and coordinates for the centre of each pore (monomers A to D), at the ar/R SF, were used to generate the pore radius along the z-axis, for each simulation.

To test the water permeation, 6 MD simulations were performed, 3 for pH 7.0 and 3 for pH 5.0. Simulations were run for 10,000,000 steps or 20,000 ps (20 ns), with a time-step of 2 fs, using the same two model systems and parameters. For these runs, the pull code was omitted, therefore removing any biasing of the system. Water molecules were counted using a python script, based on a Tcl script for tracking water molecules in a simulation [54], to determine each water molecule’s position at each step and track its progress over the simulation time, registering full passage through the tetramer as well as the direction of movement. The upper and lower limits on the tetramer height were taken from the pore radius data calculated using the HOLE 2.0 program [53]. Visualization of the trajectories and Hole 2.0 pore surfaces and sizes was performed using VMD (https://www.ks.uiuc.edu/Research/vmd).

### 2.10. Water and Glycerol Permeation from Molecular Dynamics

Glycerol movement across hAQP7 was monitored by analysing its trajectories for the whole duration of the simulations. For each simulation, glycerol molecules able to completely cross the hAQP7 channels were accounted for and all others excluded. The final data is represented as the mean ± SEM of total glycerol molecules crossing the aquaporin channels.

Due to the fact that the molecular dynamics used in this work represents an equilibrium simulation, only the diffusion constant (D_w_) can be estimated, rather than the permeability coefficient. For this purpose, D_w_ of the single-file water molecules was estimated using the Einstein relation [55,56], as described by Horner et al. [57]: D_w_ = k_o_z^2^/2, where *z* represents the average distance between two water molecules in the single-file region and *k*_o_ represents the transport rate. The transport rate was calculated as the total number of water molecules crossing the channel along the Z-axis, given by the script, divided by the length of the simulation (in seconds). The final data is represented as the mean ± SEM.

### 2.11. Statistical Analysis

The results were expressed as mean ± SEM of *n* individual experiments. Statistical analysis between groups was performed by the unpaired Student’s *t*-test using the Prism software (GraphPad Software Inc., San Diego, CA, USA). *p* values < 0.05 were considered statistically significant.

## 3. Results

### 3.1. hAQP7 Is a Functional Water and Glycerol Channel When Expressed in Yeast

In this work, we investigated hAQP7 activity and pH regulation in a *S. cerevisiae* model, previously optimized by us and used for heterologous aquaporin functional studies [17,19,25,32,58]. Yeast cells were transformed with either the empty plasmid (control cells) or the plasmid containing the human AQP7 gene (mentioned as hAQP7 cells for clarity). Expression and subcellular localization of hAQP7 at the yeast plasma membrane were confirmed by fluorescence microscopy using GFP-tagging (Figure S1A).

**Figure 1:**
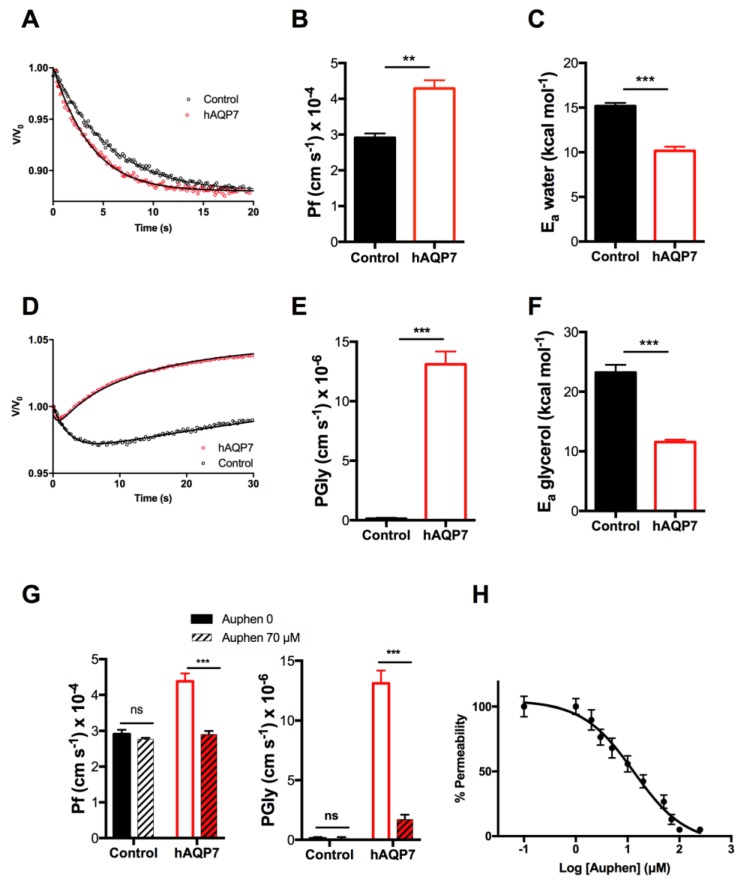
Water and glycerol permeability in control and cells expressing hAQP7. (**A**) Representative time course of the relative cell volume (*V*/*V*_0_) changes after a sorbitol hyperosmotic shock (pH 7.4, 23 °C) inducing water efflux and cell shrinkage. (**B**) Water permeability coefficient (*P*_f_) and (**C**) activation energy (*E*_a_) for water permeation. (**D**) Representative time course of the relative cell volume (*V*/*V*_0_) change after a glycerol hyperosmotic shock (pH 7.4). After the fast water outflow, glycerol influx induces cell reswelling. (**E**) Glycerol permeability (*P*_Gly_) and (**F**) activation energy (*E*_a_) for glycerol permeation. (**G**) Water and glycerol permeability of cells upon treatment with Auphen (30 min, 70 μmol/L). (**H**) Dose-response curve of glycerol permeability inhibition by Auphen, IC_50_ = 12.95 ± 0.35 μmol/L. All permeability assays were performed at 23 °C, except for E_a_ assessment. Data are shown as mean ± SEM of five independent experiments. ns, non-significant; ** *p* < 0.01; *** *p* < 0.001.

The activity of hAQP7 was assessed by stopped-flow fluorescence spectroscopy by challenging cells equilibrated in isosmotic solution at pH 7.4 (mammalian physiological pH) with hyperosmotic solutions of sorbitol (impermeant solute, inducing water efflux and cell shrinkage) or glycerol (after the initial fast water outflow, glycerol influx induces cell re-swelling [15]). Yeast expressing hAQP7 showed faster volume equilibration and higher osmotic permeability coefficient *P*_f_ ((4.38 ± 0.23) × 10^−4^ cm·s^−1^) than control cells ((2.91 ± 0.12) × 10^−4^ cm s^−1^) (Figure 1A,B), evidencing water channeling. Regarding glycerol permeability, *P*_Gly_, a marked difference in the volume change rate between control and hAQP7 cells is observed (Figure 1D), where the permeability, *P*_Gly_, for hAQP7 cells was observed to be approximately 100-fold the control ((13.1 ± 1.80) × 10^−6^ and (0.12 ± 0.07) × 10^−6^ cm s^−1^ for hAQP7 and control cells, respectively) (Figure 1E). To confirm the contribution of hAQP7 for the observed *P*_f_ and *P*_Gly_, the activation energies (*E*_a_) for both water and glycerol permeation were measured (Figure 1C,F). *E*_a_ is a crucial parameter that allows distinction between passive bilayer diffusion and protein-mediated diffusion through membranes. The observed *E*_a_ was lower in hAQP7 cells (9.16 ± 0.45 and 10.55 ± 0.41 kcal·mol^−1^ for water and for glycerol) when compared to control cells (15.06 ± 0.46 and 23.20 ± 1.31 kcal·mol^−1^ for water and for glycerol), indicating that hAQP7 expressed in yeast is a functional water and glycerol channel.

Previous studies by our group have detected the strong and selective inhibitory effect of a gold(III) coordination compound, Auphen [Au(phen)Cl_2_]Cl (phen = 1,10-phenanthroline), on hAQP3 and hAQP7, expressed in human erythrocytes and mammalian cultured cells, respectively [34,35,59,60]. Here, we used Auphen to validate hAQP7 activity expressed in yeast and demonstrate that it is possible to detect small changes in channel permeation. Auphen induced a marked inhibitory effect of hAQP7 permeability (34% and 84% for *P*_f_ and *P*_Gly_, respectively) (Figure 1G). Additionally, a dose-response curve for P_Gly_ inhibition by Auphen resulted in an inhibitory concentration (IC_50_) of 12.95 ± 0.35 μmol/L (Figure 1H), in line with previously reported studies in hAQP7 expression in cultured adipocytes [35].

### 3.2. hAQP7 Is a pH-Sensitive Water and Glycerol Channel

hAQP7 pH-dependency has been previously evaluated in Madin-Darby canine kidney (MDCKII) cells by measuring ^14^C-glycerol uptake in the pH range of 5.5 to 8.0 and, although glycerol uptake was significantly reduced at pH 5.0, no meaningful physiological relevance was attributed to this effect [61]. A recent study also showed pH-dependence of murine AQP7 when expressed in yeast, with a loss of glycerol permeability at pH 4 and below [62]. This prompted us to investigate the pH-sensitivity of hAQP7 using our optimized yeast-cell system where channel permeability was fully characterized as shown above.

**Figure 2:**
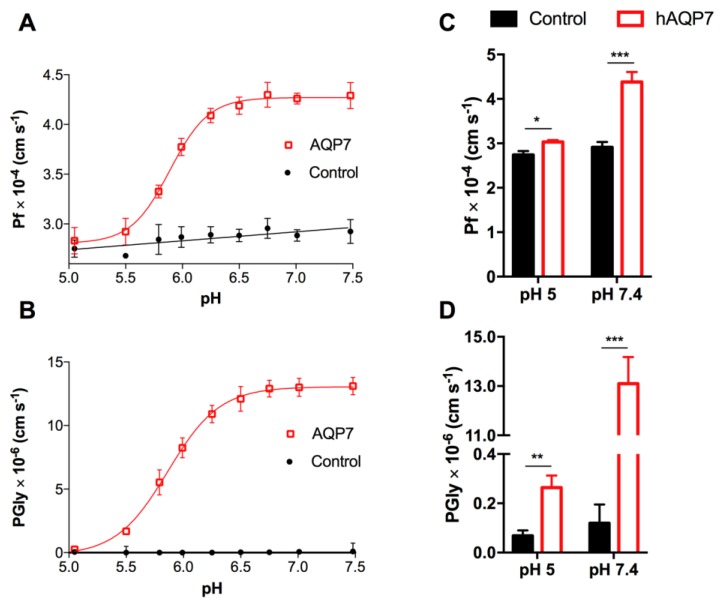
pH-dependence of hAQP7 permeability. (**A**) *P*_f_ and (**B**) *P*_Gly_ dependency of control and hAQP7 cells at pH 5.0–7.5. (**C**) *P*_f_ and (**D**) *P*_Gly_ at pH 5.0 and pH 7.4. pH-dependence of hAQP7 was analyzed by fitting the experimental data to a Hill equation from where the p*K*_a_ values were estimated. Permeability assays were performed at 23 °C. Data are mean ± SEM of four independent experiments. * *p* < 0.05, ** *p* < 0.01, *** *p* < 0.001.

Thus, *P*_f_ and *P*_Gly_ were evaluated by varying external pH from 7.4 to 5.0 (Figure 2A,B). Data shows that, while at pH 7.4, hAQP7 is fully functional and has a maximal permeability at pH 6.5 and above, and a marked reduction (more than 90% decrease) of *P*_f_ and *P*_Gly_ is observed at pH 5.0 (Figure 2C,D). Interestingly, despite this clear reduction, *P*_f_ and *P*_Gly_ were still significantly higher than control cells, indicating that although the permeability is substantially reduced, the pore is not completely closed at pH 5.0. Remarkably, the pH-dependence profile was similar for both water and glycerol permeation, with p*K*_a_ values estimated as 5.88 ± 0.01 and 5.85 ± 0.01, respectively. Additionally, the estimated *E*_a_ at three distinct pH values (pH 5.0, 6.5, and 7.5) corroborate the proposed channel regulation upon extracellular acidification (Figure S2).

### 3.3. In Silico Analysis of hAQP7 Pore Size and Permeability

**Figure 3:**
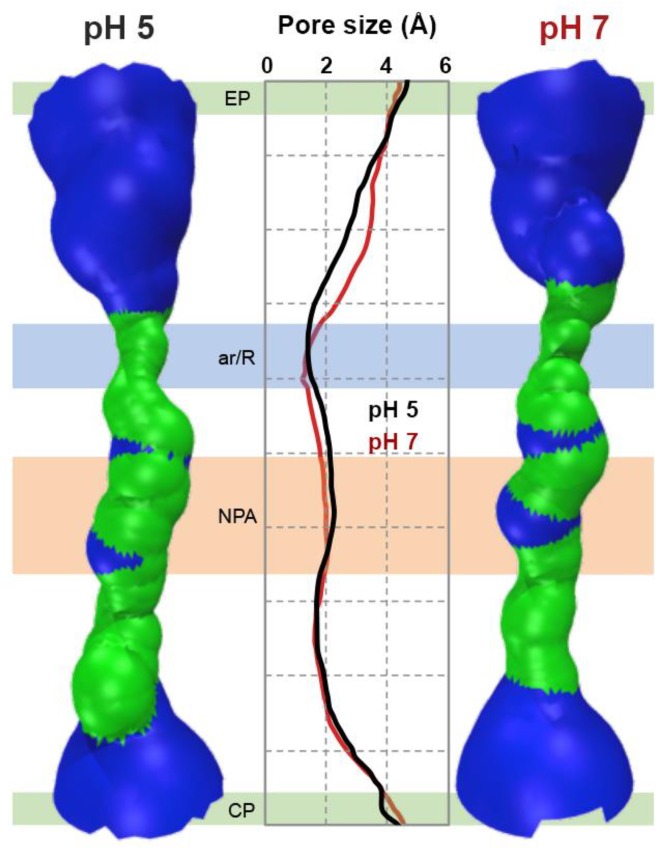
Pore size of hAQP7 monomer A at pH 5.0 and pH 7.0. Surface of a representative snapshot of monomer A (based on van der Waals radii): Red = smaller than single H_2_O, green = single H_2_O, blue = larger than single H_2_O. Pore size represented as an average of all monomers in five simulations at each pH value (pH 5.0 in black and pH 7.0 in red traces). EP–extracellular pocket, ar/R–aromatic/arginine selectivity filter, NPA–NPA motif, CP–cytoplasmic pocket. Figure generated by HOLE [53] and VMD [63].

To gain further insights into hAQP7’s pH regulation, we used a molecular modelling approach previously developed by our group [32]. A homology model of hAQP7’s tetramer was obtained using MOE software (MOE 2012.10; CCG 2012) [45], and based on the available structure of *E. coli’*s glycerol facilitator (GlpF, pdb code 1FX8) [7]. In each protein monomer, the common fold shared by the aquaporin family was found: Six transmembrane helices and two half-helices, with their N-terminal ends located in the centre of the pore (NPA motif). However, hAQP7 has NAA (Asn94, Ala95, Ala96) and NPS (Asn226, Pro227, Ser228) motifs, rather than the common NPA found in other AQPs. A second selectivity filter, located near the extracellular entrance, named aromatic/arginine selectivity filter (ar/R SF), is an important distinctive and conserved feature of the aquaporin family. This is observed in our model of hAQP7, where Phe74, Tyr223, and Arg229 constitute the ar/R SF. The refined homology model of hAQP7 was protonated using the PROPKA 3.1 package [47] at pH 5.0 and 7.0 and five independent MD simulations were run for 0.5 ns for each pH system (pH 5.0 and 7.0). To investigate possible pore closure and conformational changes at low pH, the size and shape of the pore were analysed using HOLE [53], from snapshots taken at five time points in each MD simulation. In Figure 3 is shown a surface representation of channel A, next to the average channel size of all monomers throughout the simulations. Interestingly, no differences were found in the size of the channels at the two pH values for both NPA and ar/R SF areas, indicating that the decrease in permeability may not be due to conformational changes and pore closure. However, there is an observable difference in the extracellular pocket (EP) (0.5 Å broader), just above the ar/R SF. This small variation may be due to system fluctuation during the simulation (as previously observed for other aquaporin isoforms [16]), as it was not detected in all individual cases (Figure S3A). Yet, such conformational difference at pH 7, leading to a broader extracellular glycerol binding pocket just above the ar/R SF, could favour its passage through the pore, leading to increased permeability.

**Figure 4:**
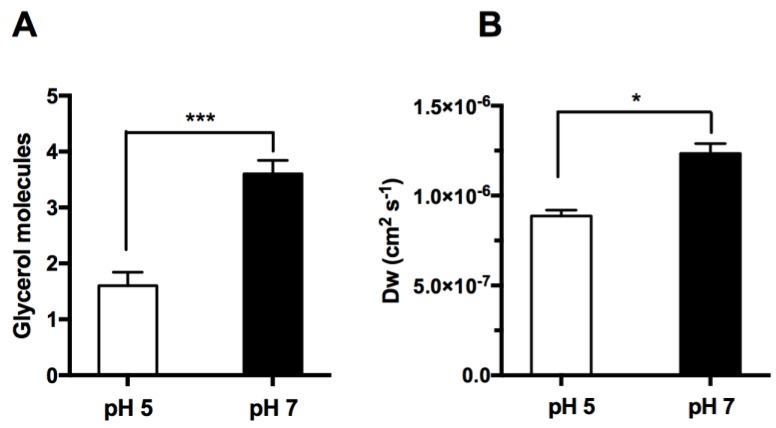
pH dependence of glycerol and water permeation across the hAQP7 channel (in silico). (**A**) Number of glycerol molecules crossing the AQP7 tetramer in MD studies, represented as mean ± SEM (n = 5). (**B**) Water diffusion coefficient calculated by MD simulations, represented as mean ± SEM (n = 3). n = number of simulations. * *p* < 0.05, *** *p* < 0.001.

Since conformational analysis of the pore indicates that the protein does not alter pore size when protonated at pH 5.0 (Figure 3), we decided to investigate if glycerol and water fluxes were affected in silico. Hence, five independent MD simulations were run for 0.5 ns for each pH (pH 5.0 and 7.0) with four glycerol molecules, located in the extracellular side of the membrane, pulled along the Z-axis towards the intracellular space, mimicking imposed glycerol gradients. A total of 10 simulations were run to evaluate glycerol permeability (see supplemental material for videos of the MD simulations). Afterwards, all simulations were analysed and glycerol molecules monitored for successful permeation of hAQP7. In Figure 4A, glycerol permeation is shown as an average number of glycerol molecules able to cross the tetramer via monomer channels. As expected, glycerol permeability is significantly higher at pH 7.0 than at pH 5.0, corroborating the obtained experimental *P*_Gly_ (Figure 2B).

For water permeation, longer simulations (20 ns) were run at both pH values, without any pull code. The movement of water molecules was evaluated using a script developed by us and based on previous work [54]. For this purpose, only water molecules that completed the crossing through the AQP7 channel were counted, excluding all molecules crossing via the lipid bilayer. Water diffusion (*D*_w_) was calculated as described in the experimental section and based on reference [57], simulating an equilibrium situation. As depicted in Figure 4B, *D*_w_ evaluated at pH 7.0 was significantly higher than at pH 5.0.

In conclusion, although the MD and stopped-flow spectroscopy data may not be directly comparable since the computational studies are not performed in osmotic conditions, our in silico and in vitro data are in accordance and demonstrate that glycerol and water permeation by hAQP7 are pH-regulated.

### 3.4. Tyr135 and His165 Are Key Residues for Glycerol Permeability

Previous research on human AQP3 using site-directed mutagenesis [30] demonstrated that four residues (His53, Tyr124, Ser152, and His154) appear to be responsible for pH gating of this isoform. The relevance of these residues on hAQP3 was recently investigated by our group using MD and we proposed that gating occurs mainly due to protonation of His154 and its interaction with the neighboring His129 [32].

**Figure 5:**
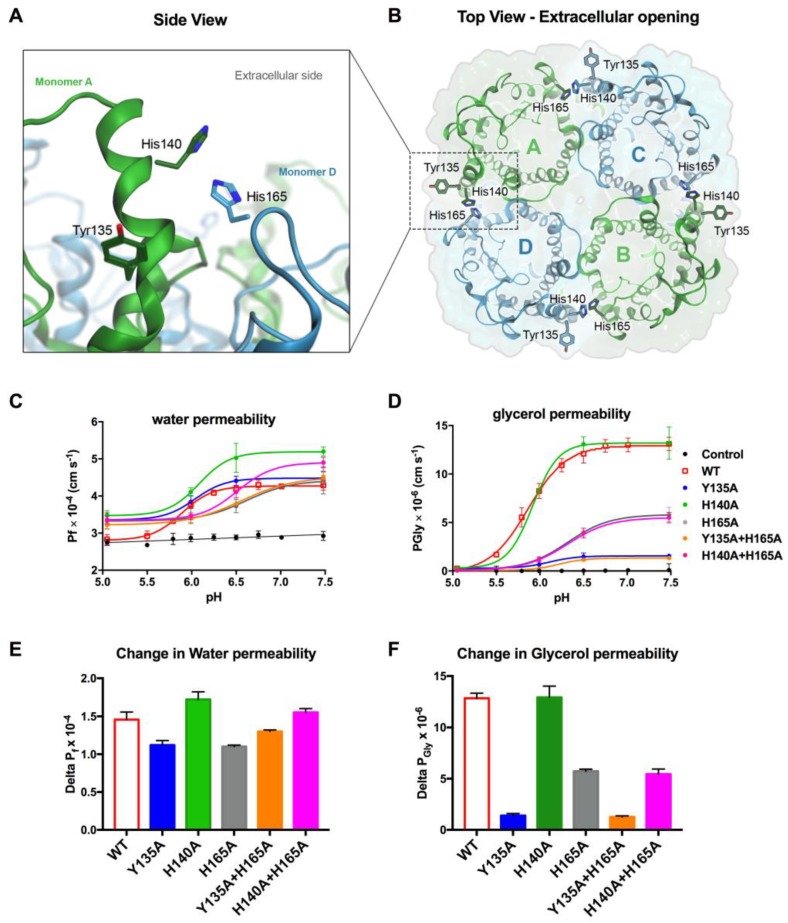
Putative residues involved in hAQP7 pH regulation. (**A**) Side and (**B**) top views of the refined homology model of human AQP7 in its tetrameric assembly, in cartoon representation of the tertiary structure, with ribbon representation of each monomer. Amino acid residues are shown in stick representation and backbone and hydrogens are hidden for clarity. Residues are colored according to the corresponding monomer, as shown in B. Figure generated with MOE [45]. (**C**) Water permeability (*P*_f_) and (**D**) glycerol permeability (*P*_Gly_), measured at pH 5.0 to 7.5, of control cells and cells expressing hAQP7-WT and -mutants. (**E**) Magnitude of the change in water (Delta Pf) and in (**F**) glycerol (Delta PGly) permeability (from pH 7.4 to pH 5.0) due to point mutations. All permeability assays were performed at 23 °C. Data (mean ± SEM, n = 4 for each data set) were fitted according to the Hill equation.

To investigate the mechanism of pH regulation, sequence alignment of hAQP3 and hAQP7 and superposition of the two homology models (the homology model of hAQP3 was prepared as described in reference [32]) showed that the corresponding residues in hAQP7 are Tyr64, Tyr135, His140, Pro163, and His165. The location of residues, Tyr135, His140, and His165, in the hAQP7 tetramer is shown in Figure 5A,B.

Detailed analysis of the protonation sites at both pH values revealed that only four residues are protonated at pH 5.0, which are not at pH 7.0 (Figure S4): On the extracellular side, His140 (in all monomers); and intracellularly, Glu40 (monomers A, C, and D), His92 (monomers B, C, and D), and Glu202 (in all monomers). Based on the sequence/structural alignment and protonation studies, the residues facing the extracellular environment chosen for further site-directed mutagenesis studies were Tyr135, His140, and His165. Additionally, as seen in Figure 3, changes in pore size are only observed in the extracellular side at pH 7.0, thus pointing at the role of extracellular amino acids in pH-dependency. Since Pro163 does not have a protonable side chain, and Tyr64 was not protonated at pH 5.0, these residues were not further investigated.

Thus, to determine the importance of His165, His140, and Tyr135, the selected amino acids were substituted by alanine residues (Ala) and, due to the proposed role of His165 on AQP3 pH gating [32], double mutants were also generated. The relative membrane expression levels revealed that all the point-mutants retained the same cellular distribution in the plasma membrane as the wild type AQP7 protein (WT) (Figure S1B). Moreover, no differences in membrane abundance were observed in cells incubated in media with different pH or osmolarity. The permeability of the mutants was then further investigated under the same pH range as previously used for WT.

Data shows that the water permeability of all mutants remains equal or even slightly higher than WT above pH 7.0, indicating that the hAQP7 mutants are functional channels (Figure 5C,E). Regarding glycerol permeation, while the single H140A mutant displayed similar behaviour to WT, all the other mutations showed markedly reduced glycerol permeability at pH 7.4 (Figure 5D,F). In fact, the mutants, H165A and H165A+H140A, reduced glycerol maximal permeability at pH 7.4 to 44% of the WT, inducing a shift in the pK_a_ from 5.85 ± 0.01 to 6.30 ± 0.02. Interestingly, the mutants, Y135A and Y135A+H165A, rendered the protein almost inactive, reducing the maximal P_Gly_ to 10% of the observed for WT (Figure 5D,F). This suggests the key involvement of the Tyr135 and His165 residues in the channel pore permeability, while His140 alone does not appear to have any contribution to pH-dependency.

### 3.5. Protonation of hAQP7 Induces Changes in Protein Surface Electrostatic Charges

Following the functional in vitro results showing the involvement of Tyr135 and His165 in hAQP7 permeability, detailed analysis of the MD trajectories was performed, but a clear role for these residues in pH-gating was not evident. However, careful analysis of the hydrogen bond network during glycerol permeation at pH 7.0 shows that glycerol preferably forms hydrogen bonds with residues featuring protonable side chains (e.g., Lys63, Tyr64). Protonation of residues in the pore entrance (such as His165) at pH 5.0 may alter the hydrogen bond network, also affecting the residues responsible for glycerol passage. Therefore, we postulate that the contribution of His165 and Tyr135 to AQP7 pore permeability is based on a network of hydrogen bonds that dynamically readapts to changes in external acidification. In order to support this hypothesis, we prepared new homology models for all the mutants. The models were generated based on mutated sequences, refined and subsequently protonated at pH 5.0 and pH 7.0, as performed for the WT model. Each model was further analysed for pore size (Figure S3B).

**Figure 6:**
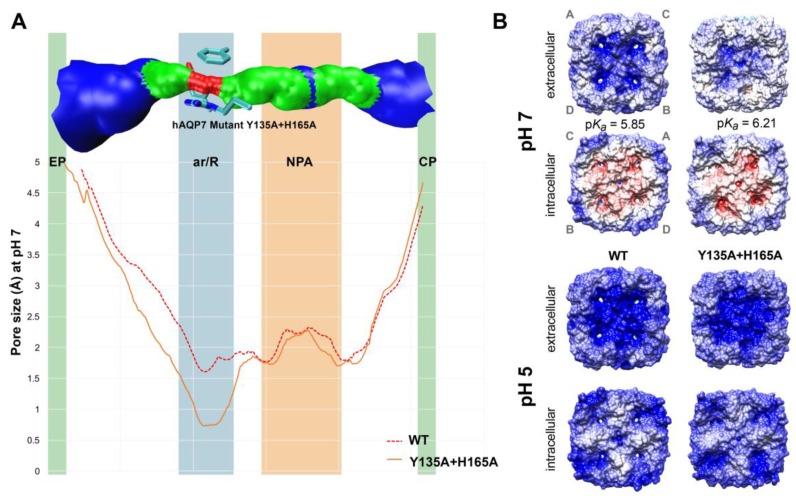
Pore size and protein surface electrostatic charges. (**A**) Pore size of human AQP7-WT and double mutant Y135A+H165A, analyzed using HOLE [53]. Top channel surface represents the pore size of hAQP7 mutant, Y135A+H165A (based on VDW radii): Red = smaller than single H_2_O, green = single H_2_O, blue = larger than single H_2_O. Figure generated by HOLE [53] and VMD [63]. (**B**) Electrostatic surfaces of hAQP7-WT and mutant Y135A+H165A, at pH 5.0 and 7.0, with the following color code: Positively charged = blue, negatively charged = red, neutral = white. Top views of both intra and extracellular sides, with the p*K*a of each protein indicated in the figure. Surfaces were generated using the Adaptive Poisson-Boltzmann Solver (APBS) [48] plugin in Chimera [49]. Monomers labeled A–D are shown in grey to indicate their position in each view.

The main difference in size was found for the double mutant, Y135A+H165A (Figure 6A), which showed a decrease in pore size at the ar/R SF of approximately 1 Å when compared to the WT. All mutants show a pore size decrease in this region (Figure S3B), although not as marked (ca. 0.5 Å). Moreover, none of the mutants show relevant changes in size in the NPA region. Instead, a decrease in the size of the ar/R SF, the first constriction site that solutes encounter when permeating AQPs from the extracellular side, may contribute to the observed loss of permeability of the mutants. However, His140A also shows a slight decrease in ar/R SF size, despite having the same permeability of the WT protein. This indicates that pore size may not be the only factor playing a role in AQP7 permeability. In fact, as observed in Figure 5 and Table S2, the studied mutants also appear to alter the p*K*_a_ of water and glycerol permeation. This effect may be due to changes in the electrostatic surface of the mutants and also under different pH conditions. In fact, electric fields within protein pores have been identified as barriers to solute passage [64]. Therefore, in order to study the effects of pH and mutations in the electrostatic surface of hAQP7, surfaces were generated using the Adaptive Poisson-Boltzmann Solver (APBS) [48] plugin in Chimera [49], and are shown in Figures 6B and S5.

Overall, the main observable difference is that the intracellular electrostatic surface of all models appears to be much more negative (shown in red colour) at pH 7.0 than at pH 5.0 (Figure 6B). At pH 7.0, the inner channel areas appear more electrostatically positive. Interestingly, the H140A mutant is very similar to WT, both in electrostatic distribution on the protein surface at both pH values and in terms of protonation (Figure S5), which is in line with our experimental findings.

The most marked changes were observed for the H165A and Y135A single and double mutants, pointing to the crucial involvement of these two residues in pH gating and glycerol permeability. Remarkably, as observed for glycerol permeability, Y135A mutation has the strongest effect on the double mutant’s surface, also shown to have the same protonated residues as the single mutant (Figure S6). Similarly, H165A mutation appears to dominate the effects of the double His mutant, both in permeability and residue protonation.

Analysis of protonation at both pH values reveals that the mutations do not induce changes in protonation of the intracellular residues, Glu40 and Glu202. However, at pH 5.0, the other protonable intracellular site, His92, is deprotonated in the mutants, H165A and H140A+H165A, and partially deprotonated in the H140A mutant. Changes in protonation of this residue do not appear to affect the permeability at the lowest pH of any of the mutants, indicating that deprotonation of His92 is not a major player in the permeation mechanism. Despite the fact that none of the mutants shows protonation changes of intracellular residues at pH 7.0, it is possible to observe that the intracellular charge distribution is altered in Y135A, H165A, and in the double mutants: The electrostatic surface of the channel is more negative than WT. This indicates that changes in extracellular residues affect the overall electrostatic surface of the protein, including the intracellular surface side. Thus, changes in extracellular pH may be able to affect the intracellular charge distribution, likely by altering the H-bond network of the protein, thus, affecting the permeability.

Moreover, the pK_a_ differences appear to be due to the changes in the electrostatic protein surface, caused by the mutations, which can alter the protein’s affinity for substrates. Yet, mutating these residues does not render the protein pH-insensitive. Interestingly, beside the residues mutated in this study, most of the protonable amino acids composing the protein surface are lysines and arginines, with pK_a_ above 10, making them very unlikely players in pH-sensitivity in a physiological range. A likely scenario is that the protein rearranges its H-bonding network according to the newly inserted residues, redistributing the electrostatic potential and changing affinity for substrates, shifting the role of pH-sensitivity to other nearby residues. The modulation of transport through electrostatic is quite typical of biological channels and has been previously observed for different porins [64].

## 4. Discussion

Aquaglyceroporins have emerging roles in energy metabolism and adipose tissue homeostasis and have been implicated in obesity and metabolic-related complications, such as metabolic syndrome (reviewed in [6]). AQP7 is the main glycerol channel expressed in human adipose tissue, with a fundamental role in glycerol release from adipocytes during lipolysis, when triglycerides are hydrolysed to fatty acids and glycerol [65,66]. During fasting, the intracellularly produced glycerol is channelled by AQP7 into the bloodstream to be taken up in the liver by AQP9 where it will be used for gluconeogenesis. Thus, the fine-tuning of AQP7’s efficiency for glycerol release from the adipose tissue and its uptake by the liver might be the rate-limiting step for the maintenance of normal blood glucose levels. While AQP7 pH-sensitivity has been previously assessed, the molecular basis of pH-regulation has never been elucidated.

This study allowed disclosing the mechanism of pH regulation of human AQP7 activity at a molecular level, showing that this channel changes from a fully active to nearly inactive state when the pH drops from 7.5 to 5.0. Using in vitro and in silico approaches, we found that residues, Tyr135 and His165, facing the extracellular environment are crucial for channel permeability and we hypothesize that hAQP7’s lack of glycerol permeability may be due to changes in the overall hydrogen bond network rather than a truly open/closed pore mechanism of gating. Furthermore, due to the position of the key pH sensitive residues at the monomer-monomer interface, it is possible that a cooperativity change between the AQPs monomers within the tetramer structure affects permeability, as observed for other aquaporins [67,68].

According to our experimental data, the maximum of protein function is achieved at the pH range of 6.7 to 7.5. In fact, knowing that hAQP7 activity is crucial for glycerol efflux from adipose tissue to be used for gluconeogenesis in the liver, full activity of glycerol channel permeation at a mammalian physiological pH range would be expected. The observation that hAQP7 still retains 50% permeability above pH 6.00 is very interesting. Notably, during intracellular lipolysis, breakage of triglycerides into glycerol and free fatty acids (FFA) have been shown to release protons, acidifying the intracellular pH in a stoichiometric manner [69,70]. However, in mature adipocytes after stimulation with lipolytic agents, the intracellular pH does not drop below 6.5 [71,72], implying that AQP7 pH sensing may not affect glycerol efflux. However, it is possible that significant levels of lipolysis with increased H^+^ released into the adipose micro-circulation create local acidic microenvironments, causing metabolic acidosis [69,70]. In fasting conditions, when lipolysis occurs, AQP7 has a role in facilitating glycerol efflux from the adipocytes into the bloodstream, that will then be taken up by liver AQP9 for gluconeogenesis. It is plausible that AQP7’s ability to prevent extracellular glycerol influx at local acidic conditions while the efflux is maintained during lipolysis contributes to its physiological role in the regulation of energy homeostasis. Notably, adipocytes also express other aquaglyceroporins that account for glycerol efflux, namely AQP3 [73] and AQP10, the latter being considered as an alternative pathway for glycerol efflux [74]. An opposite pH gating from AQP3 and AQP7, with low permeability at physiological pH and higher permeability at acidic pH, was recently disclosed for AQP10 that displays a unique cytoplasmic glycerol-specific pH-dependent gating mechanism [72]. Thus, it is possible that AQP10 stimulation during lipolysis coordinates with AQP7 and AQP3 to assure efficient glycerol efflux important for controlling body fat mass. Moreover, the fact that AQP7 is also present in the small intestine [75], kidney [76], endocrine pancreas [77,78], capillary endothelia [79,80], and heart and skeletal muscle [81,82], where other aquaglyceroporins are also expressed [83], suggests that a coordinated regulation of glycerol fluxes is in place to assure fine-tuning of energy homeostasis.

In addition, expression of AQP7 in testis [84] and in human sperm cells [85,86] has been reported, and was correlated with sperm motility and viability [85,86,87], suggesting that AQP7 has some role in sperm energy metabolism and late spermatogenesis. Indeed, glycerol is a potential substrate for spermatozoa during their passage through the epididymis and may be utilized as an energy substrate in spermatogenesis [87,88,89], probably channeled by AQP7. A recent study investigated cultured spermatozoa motility and capacitation at a pH range of 5.2 to 8.2, and concluded that acidic pH results in impaired sperm motility [90]. Also, the alteration of pH that not only occurs in male semen, but also in the female vagina under infections, can have a profound effect on sperm motility and quality and even result in infertility [90,91]. Therefore, it is tempting to speculate that the pH regulation of human AQP7 has biological relevance for sperm maturation and quiescence and may be involved in fertility.

Overall, considering the importance of glycerol in multiple vital physiological processes, regulation of its permeation across hydrophobic cell membranes via AQPs may be crucial for energy homeostasis, with consequences for cell proliferation, adaptation, and survival. Thus, the mechanisms involved in the regulation of hAQP7 by acidification provide new hints for the design of new molecules targeting aquaglyceroporins and opens new perspectives for the treatment of disorders affected by glycerol unbalance.

## Supplementary Materials

The following are available online at http://www.mdpi.com/2073-4409/7/11/207/s1. Figure S1: Localization of GFP-tagged hAQP7 expressed in *S. cerevisiae* aqy-null strain, Figure S2: Activation energy (*E*_a_) for water and glycerol permeation at distinct pH values, Figure S3: Pore size of AQP7 monomers at pH 5.0 and pH 7.0, Figure S4: Extracellular and intracellular views of the homology model of human AQP7, Figure S5: Electrostatic surfaces of hAQP7 WT and mutants, at pH 5.0 and 7.0, Figure S6: Sequence alignment of hAQP7 WT and mutants, Table S1: Human AQP7 specific primers for PCR amplification and mutagenic primers used in this study, Table S2: pH-dependency (p*K*_a_ values) of water and glycerol permeation of human AQP7 (wild-type and mutated), Video S1: Permeation of glycerol via hAQP7, embedded in a lipid bilayer, at pH 7.0, Video S1: Permeation of glycerol via hAQP7, embedded in a lipid bilayer, at pH 5.0.
