# Supplementary material for: Molecular Basis of Aquaporin-7 Permeability Regulation by pH
Source: Cells. 2018 Nov 10;7(11):207. doi: 10.3390/cells7110207 (PMC6262577; doi:10.3390/cells7110207)
Supplement: Supplementary file 1 [file cells-07-00207-s001.zip › SI AQP7 Cells MDPI.pdf]

Supplementary Material

Molecular basis of aquaporin-7 permeability regulation by pH

Andreia F. Mósca[1,2#], Andreia de Almeida[3#], Darren Wragg[3], Ana P. Martins[1,2], Farzana Sabir[1,4], Stefano Leoni[3], Teresa F. Moura[1,5], Catarina Prista[4], Angela Casini[3*], Graça Soveral[1,2*]

Table S1 – Human AQP7 specific primers for PCR amplification and mutagenic primers used in this study.
Restriction sites for *Spe*I and *Cla*I are in bold and mutated nucleotides introduced are underlined. Each mutation was confirmed by DNA sequencing to ensure the fidelity of PCR reactions.

Primer name	***Sequence (5' to 3' direction)***
**Cloning**	
AQP7_FW	GG**ACTAGT**CCTATGGTTCAAGCATCCGGGCACAG
AQP7_REV	CC**ATCGAT**GGAGAAGTGCTCTAGGGCCATGGATTCAT
**Site-directed mutagenesis**	
Tyr135Ala_FW	GCTGCCACCATCTACAGTCTCTTC<u>GCC</u>ACGGCCATTCTCCACTTTTCG
Tyr135Ala_REV	CGAAAAGTGGAGAATGGCCGT<u>GGC</u>GAAGAGACTGTAGATGGTGGCAGC
His140Ala_FW	CTCTTCTACACGGCCATTCTC<u>GCC</u>TTTTCGGGTGGACAGCTGATGGTG
His140Ala_REV	CACCATCAGCTGTCCACCCGAAAA<u>GGC</u>GAGAATGGCCGTGTAGAAGAG
His165Ala_FW	GCCACCTACCTTCCTGAT<u>GCC</u>ATGACATTGTGGCGGGGCTTCCTG
His165Ala_REV	CAGGAAGCCCCGCCACAATGTCAT<u>GGC</u>ATCAGGAAGGTAGGTGGC

Table S2 – pH-dependency (pK_a values) of water and glycerol permeation of human AQP7 (wild-type and mutated). Data presented as mean ± SEM (n=4). *p < 0.05 vs wild-type.

	Water	***Glycerol***
	pK_a	pK_a
Wild-type	5.89 ± 0.02	5.85 ± 0.01
Y135A	6.06 ± 0.01	6.04 ± 0.01
H140A	6.11 ± 0.02	5.91 ± 0.01
H165A	6.65 ± 0.01*	6.30 ± 0.02*
Y135A+H165A	6.58 ± 0.01*	6.21 ± 0.02*
H140A+H165A	6.53 ± 0.01*	6.29 ± 0.01*

Values were compared to AQP7 wild type. *, P<0.05

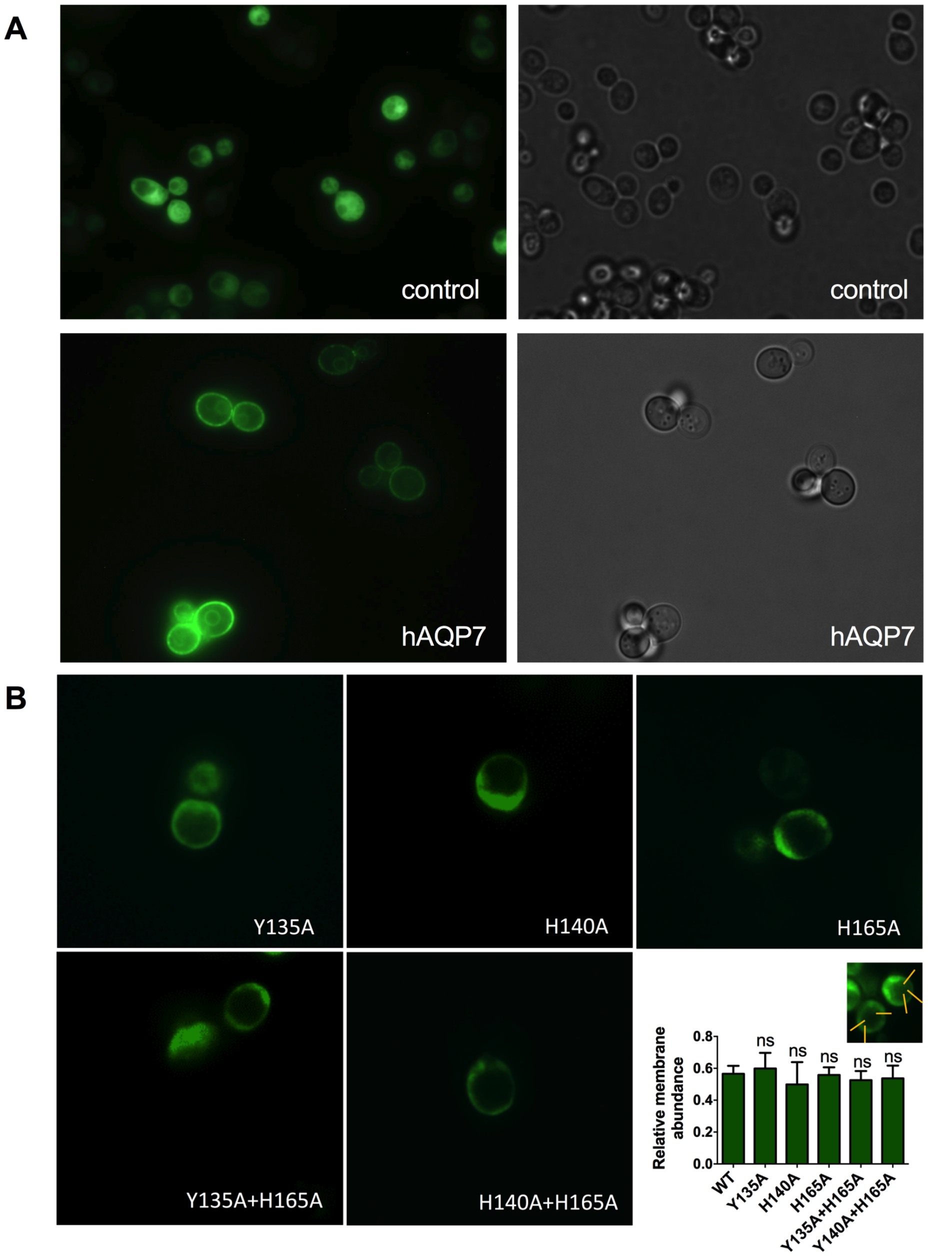

Figure S1 – Localization of GFP-tagged hAQP7 expressed in *S. cerevisiae* aqy-null strain.

(A) Epifluorescence (left panels) and phase contrast (right panels) images yeast aqy-null strains transformed with the empty plasmid pUG35 (control cells) and hAQP7. In control cells, GFP expression results in a homogeneous distribution of fluorescence in the cytoplasm, while yeast cells expressing hAQP7-GFP show membrane-localized fluorescence confirming hAQP7 localization at the plasma membrane.

(B) Plasma membrane localization and relative membrane abundance of GFP-tagged hAQP7 mutations. Photos are representative of cells incubated either in pH 7.4 or pH 5.1. Fluorescence was observed by fluorescence microscopy and the relative membrane abundance of each mutant (bar graph) was calculated from linear intensity profiles across cell membrane (yellow lines in the insert picture) from at least 30 cells (3 profiles lines per cell). No differences in membrane abundance were observed in cells incubated in media with different pHs or osmolarities. ns, non-significant.

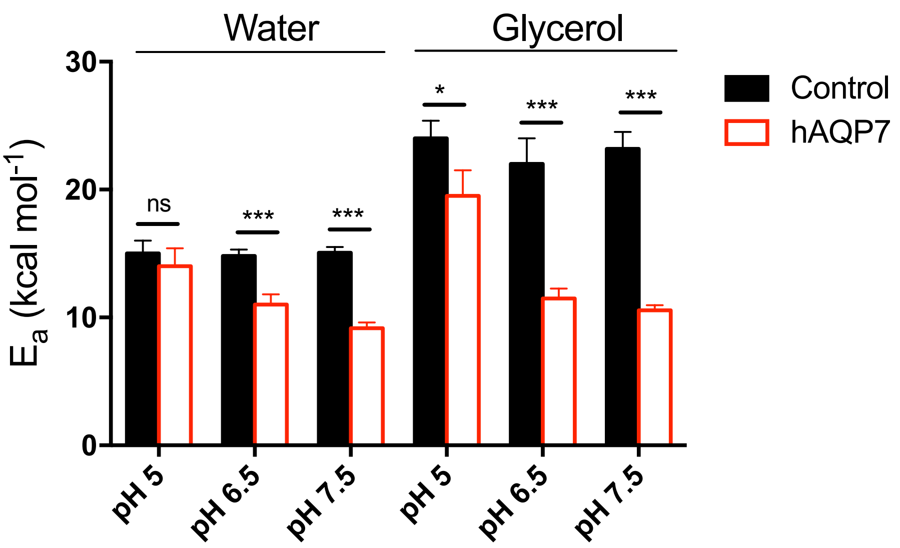

Figure S2 – Activation energy (*E_a*) for water and glycerol permeation at distinct pH values.

E_a values estimated at three distinct pH values (pH 5, 6.5 and 7.5) significantly decreased from pH 5 (water 14.0 ± 1.4 kcal mol^{-1} and glycerol 19.5 ± 2.0 kcal mol^{-1}) to pH 6.5 (water 11.0 ± 0.8 kcal mol^{-1} and glycerol 11.5 ± 0.8 kcal mol^{-1}) and pH 7.5 (water 9.2 ± 0.5 kcal mol^{-1} and glycerol 10.6 ± 0.4 kcal mol^{-1}), corroborating the proposed channel pH regulation. Data shown as mean ± SEM of three independent experiments. ns, non-significant, * $p < 0.05$; *** $p < 0.001$.

A

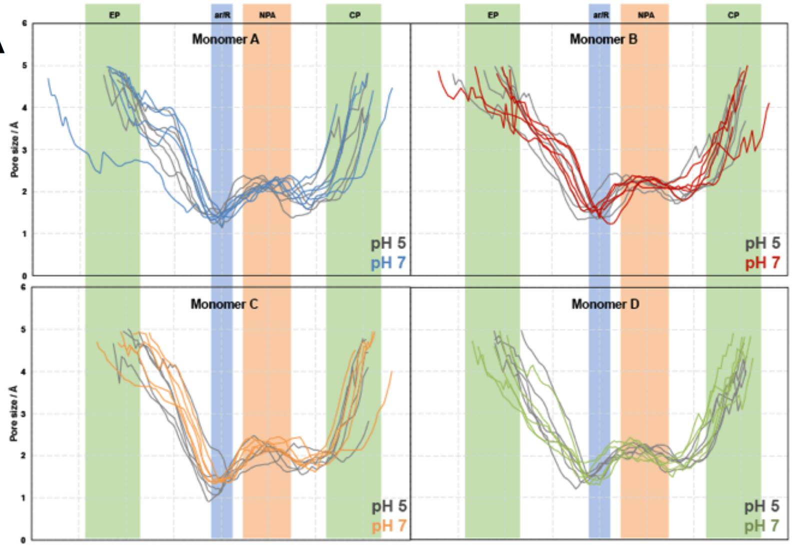

B

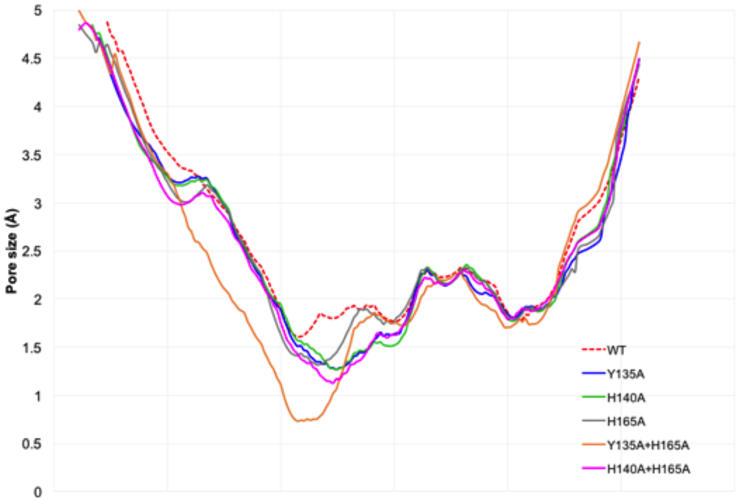

Figure S3 – (A) Pore size of the four AQP7 monomers at pH 5 and pH 7. Each line represents an average pore size for each simulation, by using the pore size taken from five snapshots of each simulation. EP – extracellular pocket, ar/R – aromatic/arginine selectivity filter, NPA – NPA motif, CP – cytoplasmic pocket.
(B) Pore size average of the four AQP7 monomers for each mutant and WT. Pore size obtained with HOLE for each homology model [1].

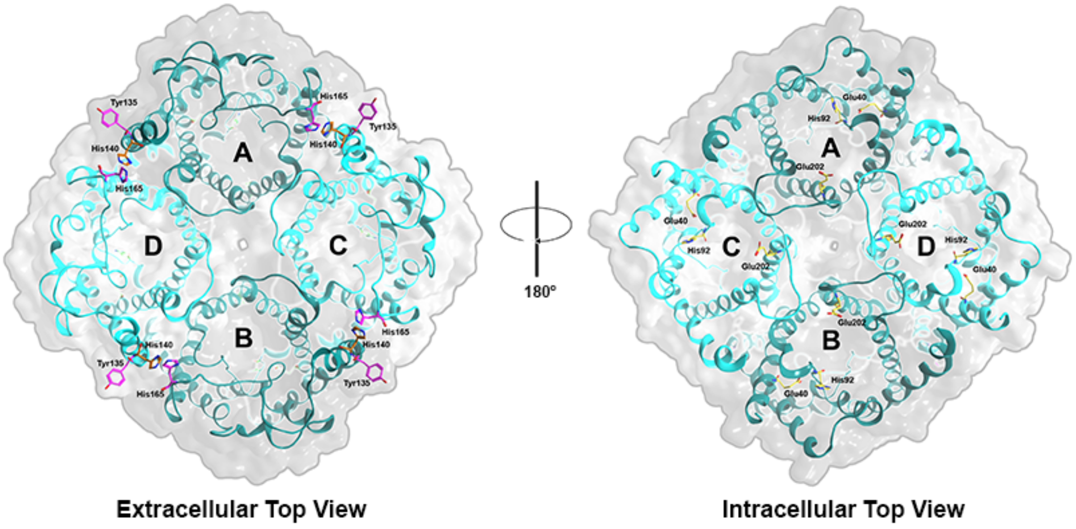

Figure S4 – (A) Top extracellular and **(B)** intracellular views of the homology model of human AQP7 in its tetrameric assembly in cartoon representation of the tertiary structure, with ribbon representation (in blue) and surface representation of residues lining the channel (grey). Relevant amino acid residues are shown in stick representation. In yellow the residues that are protonated at pH 5 in the intracellular side are shown. His140 residues in each monomer are protonated at pH 5 and shown in orange, while His165 and Tyr135 are represented in magenta; all three residues were mutated for further studies. Figure generated with MOE [2].

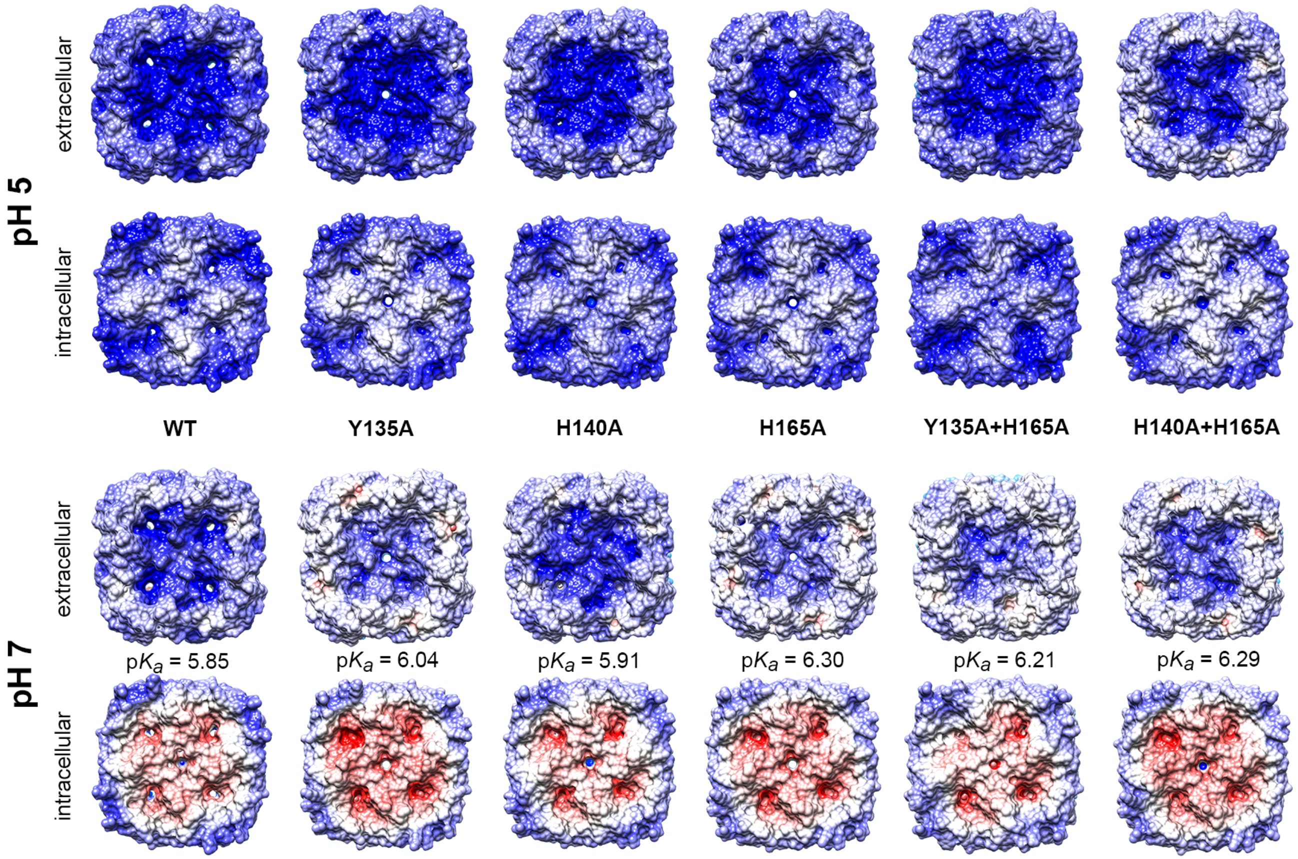

Figure S5 – Electrostatic surfaces of hAQP7 WT and mutants, at pH 5 and 7. Protonation performed using PROPKA 3.1 package [3]. Surfaces were generated using the Adaptive Poisson-Boltzmann Solver (APBS) [4] plugin in Chimera [5].

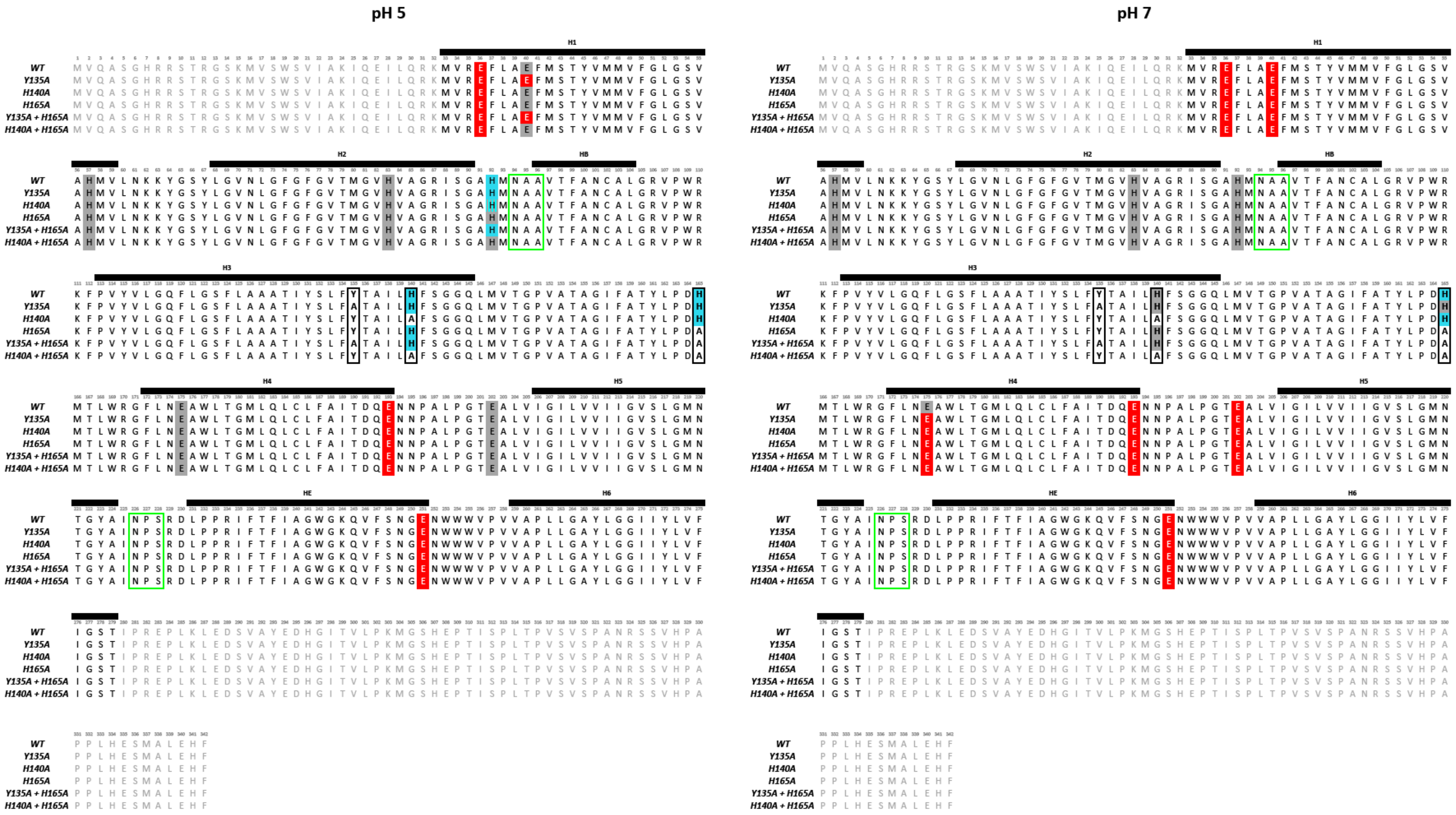

Figure S6 –Sequence alignment of hAQP7 WT and mutants. Protonation performed using PROPKA 3.1 package [3]. NPA motif shown in green boxes and mutated residues highlighted in black boxes. Charge color code: red = negative, blue = positive, grey = neutral. Residues in white do not change their protonation state under the analyzed conditions. Black horizontal bars indicate the position of the transmembrane helices (H1-6 and HB and HE).

Video S1 –Permeation of glycerol via hAQP7, embedded in a lipid bilayer, at pH 7 (AQP7 pH 7.mov).

Video S2 –Permeation of glycerol via hAQP7, embedded in a lipid bilayer, at pH 5 (AQP7 pH 5.mov).

References

1. Smart, O.S.; Neduvelil, J.G.; Wang, X.; Wallace, B.A.; Sansom, M.S. Hole: A program for the analysis of the pore dimensions of ion channel structural models. Journal of molecular graphics 1996, 14, 354-360, 376.
2. MOE, M.O.E. Chemical Computing Group Inc. Montreal, QC, Canada) 2012.10.
3. Søndergaard, C.R.; Olsson, M.H.M.; Rostkowski, M.; Jensen, J.H. Improved treatment of ligands and coupling effects in empirical calculation and rationalization of pka values. Journal of chemical theory and computation 2011, 7, 2284-2295.
4. Baker, N.A.; Sept, D.; Joseph, S.; Holst, M.J.; McCammon, J.A. Electrostatics of nanosystems: Application to microtubules and the ribosome. Proceedings of the National Academy of Sciences 2001, 98, 10037-10041.
5. Pettersen, E.F.; Goddard, T.D.; Huang, C.C.; Couch, G.S.; Greenblatt, D.M.; Meng, E.C.; Ferrin, T.E. Ucsf chimera--a visualization system for exploratory research and analysis. Journal of computational chemistry 2004, 25, 1605-1612.
